# Phenolic-Rich Wine Pomace Extracts as Antioxidant and Antipathogenic Agents Against *Pseudomonas aeruginosa*

**DOI:** 10.3390/antibiotics14040384

**Published:** 2025-04-05

**Authors:** Carolina María Viola, Mariana Elizabeth Danilovich, Romina Torres-Carro, Manuela M. Moreira, Francisca Rodrigues, Elena Cartagena, María Rosa Alberto, María Amparo Blázquez, Mario Eduardo Arena

**Affiliations:** 1 Instituto de Biotecnología Farmacéutica y Alimentaria (INBIOFAL) CONICET–UNT, Avenida N Kirchner 1900, San Miguel de Tucumán CP 4000, Tucumán, Argentina; carolinamviola@fm.unt.edu.ar (C.M.V.); mariana.danilovich@fbqf.unt.edu.ar (M.E.D.); rominatc@conicet.gov.ar (R.T.-C.); elena.cartagena@fbqf.unt.edu.ar (E.C.); 2 Facultad de Bioquímica, Química y Farmacia, Universidad Nacional de Tucumán (UNT), Ayacucho 471, San Miguel de Tucumán CP 4000, Tucumán, Argentina; 3 REQUIMTE/LAQV, ISEP, Polytechnic of Porto, Rua Dr. António Bernardino de Almeida 431, 4249-015 Porto, Portugal; manuela.moreira@graq.isep.ipp.pt (M.M.M.); francisca.rodrigues@graq.isep.ipp.pt (F.R.); 4 Departament de Farmacologia, Facultat de Farmàcia i Ciències de l’Alimentació, Universitat de València, Avd. Vicent Andrés Estellés s/n, 46100 Burjasot, Valencia, Spain

**Keywords:** wine pomace extracts, antipathogenic activity, quorum sensing inhibition, phenolic compounds, *Pseudomonas aeruginosa* virulence

## Abstract

**Background/Objectives:** Wine pomace is a rich source of bioactive phenolic compounds with potential health benefits. This study aimed to evaluate the antipathogenic and antioxidant properties of ethanol and ethyl acetate extracts from wine pomace of three grape varietals (Tannat, Bonarda, and Malbec) to explore their potential as natural alternatives for mitigating bacterial virulence in *Pseudomonas aeruginosa*. **Methods**: Successive exhaustion extractions were performed using solvents of increasing polarity (ethyl acetate and ethanol). The phenolic content was quantified, and the antioxidant activity was evaluated using standard assays. The antipathogenic activity against *P. aeruginosa* was assessed by measuring biofilm formation, elastase and protease activity, pyocyanin production, and swarming motility. Quorum sensing (QS) inhibition was tested using a violacein production assay in *Chromobacterium violaceum*. **Results:** Ethanol was more effective at extracting phenolic compounds, with Tannat exhibiting the highest total phenolic content (162.5 µg GAE/mg). HPLC-DAD analysis identified 16 phenolic acids, 18 flavonoids, and 3 stilbenes across the extracts. The ethanol extracts showed strong antioxidant activity (phosphomolybdenum reducing capacity 67–128 μg AAE/mg, ABTS^•+^ scavenging 37–71 µg/mL, Fe^3+^ reducing power 31–68 µg/mL) and inhibited biofilm formation (up to 61%), elastase (up to 41%), and protease (up to 46%) activities in *P. aeruginosa*. The extracts also reduced pyocyanin production (up to 78%) and swarming motility (up to 68%), suggesting interference with QS. Moreover, the extracts inhibited violacein production in *C. violaceum*, confirming QS inhibition (up to 26%). **Conclusions:** Among the extracts, ethanol-extracted Tannat pomace showed the most substantial antipathogenic and antioxidant activities. The results add value to wine pomace by suggesting its use as natural extracts rich in phenolic compounds, capable of controlling the bacterial virulence of *Pseudomonas aeruginosa* without promoting the development of resistance.

## 1. Introduction

Microbial contamination of foods is a major public health concern, affecting consumers, regulatory agencies, and food industries worldwide. Foodborne pathogens, including bacteria, viruses, fungi, and parasites, are responsible for millions of illnesses worldwide, with an estimated 600 million people affected annually [1]. These pathogens pose a particular risk to vulnerable populations, such as children under five, pregnant women, the elderly, and individuals with compromised immune systems. In severe cases, foodborne infections can lead to debilitating diseases, such as meningitis, and even death [1,2].

Food contamination by pathogenic microorganisms during processing and storage represents a great health and hygiene risk and produces significant economic losses for the food industry [3]. Among these microorganisms, *Pseudomonas aeruginosa* is particularly noteworthy. Commonly found in natural environments such as soil, freshwater, and marine habitats, as well as on abiotic surfaces like clinical instruments and food processing equipment [4,5], this bacterium readily attaches to food surfaces and forms biofilms. Its presence has been documented in a variety of products including dairy, meat, water, and plant-based foods [6]. Biofilm formation not only facilitates cross-contamination after processing but also complicates cleaning processes, thereby threatening human health [7].

Beyond its role in clinical infections, *P. aeruginosa* has emerged as a significant foodborne pathogen. Its rapid growth on food surfaces, coupled with the production of oxidized compounds and slimy substances, accelerates spoilage and raises concerns among consumers and food safety regulators [6,8]. Moreover, its ability to thrive in diverse environments and develop resistance to multiple antibiotics underscores the need for innovative control strategies.

*P. aeruginosa* organizes population behaviors, such as biofilm formation, swarming motility, and virulence factor production (pyocyanin, elastase, protease) by a cell-to-cell communication mechanism called Quorum sensing (QS) [9]. This mechanism allows bacteria to sense their microbial population through signal molecules named autoinducers. The most common signal molecules used by Gram-negative bacteria are *N*-acyl-homoserine lactones (AHLs) [10,11]. In many cases, the presence of these molecules would be a health risk, since they can remain in a wide variety of foods with no possibility of inactivation at 100 °C [12,13,14].

The search for QS inhibitors (QSI) is an alternative and safer approach to interrupting bacterial communication and controlling bacterial virulence factors [15,16]. Accordingly, the bioactivities of natural products like potential QSI as antipathogenic substances could be an important strategy to prevent food spoilage and foodborne diseases [17,18,19].

In this context, the food industry generates large quantities of waste and byproducts. These byproducts constitute a rich bioactive compound source, which may be applied in the food, feed, cosmetic, and pharmaceutical industries [20]. In the northwest region of Argentina, specifically Cafayate (Salta), wine production is one of the most developed activities. During wine production, tons of waste made up essentially of skins, seeds, and stems called grape pomace is generated [21,22,23]. This is characterized by a high content of polyphenol compounds that are partially extracted during the winemaking process. Generally, grape pomace contains 25–35% of the total weight of grape processed [24,25,26]. These large amounts of byproducts generated by wineries constitute a serious environmental problem [27,28].

Previous studies reported the antimicrobial activity of grape extracts against planktonic cultures of Gram-positive and Gram-negative bacteria, and the potential of grape pomace phenolic compounds to be used as preservatives [29,30,31]. However, this study seeks to interrupt bacterial communication (or QS mechanism) as an attractive strategy to current bacterial control practices employed in industrial settings [32,33,34].

This work aims to add value to red grape wine pomace as a natural alternative for preventing bacterial infections by interfering with the different QS-dependent virulence factors of *P. aeruginosa*.

## 2. Results and Discussion

*Vitis vinifera* L. is a valuable source of bioactive molecules, including lipids, proteins, carbohydrates, and polyphenols, which account for approximately 5–8% of its composition, depending on the grape cultivar [35]. Grape pomace is known for its high polyphenol content and exhibits superior antioxidant activity compared to other plant extracts [36]. Recovering *V. vinifera* pomace aligns with the principles of the circular economy, a model that promotes the reuse of products and natural resources to extend their life cycle while minimizing waste and byproducts [35].

In the present study, successive exhaustion extractions were performed on wine pomace from the three varietals using sequential extraction with ethyl acetate followed by ethanol. These solvents were used to obtain phenolic compounds of medium to high polarity, respectively. These secondary metabolites are known for their antioxidant, antimicrobial, and anti-inflammatory properties [37,38]. Therefore, they are considered ideal candidates for inhibiting pathogenic microorganisms. The composition of grape pomace extracts can vary depending on the extraction technique used [39], and factors such as geographical origin, climate, grape variety, and winemaking process further influence their phenolic composition and biological activity [40].

Several techniques have been employed to recover polyphenols from wine byproducts. The most commonly used industrial method is conventional solvent extraction, often solid–liquid or maceration, to obtain bioactive compounds from plant matrices. Water and ethanol are the solvents most employed for food, pharmaceutical, and cosmetic applications due to their safety and effectiveness [41]. The choice of solvent significantly influences the quantity and distribution of extracted phenolic compounds, affecting both the antimicrobial and antioxidant activities of the extracts [42].

Not all phenolic compounds can be efficiently isolated using a single solvent due to their varied polarities and solubilities. Employing a sequential extraction approach overcomes these limitations by gradually increasing the polarity of the solvent. This strategy enables the recovery of a broader range of polyphenols, resulting in a more comprehensive profile of bioactive compounds. Furthermore, it simplifies compound identification by reducing the number of compounds extracted per solvent, which improves the efficiency of analytical techniques [43,44,45].

In this work, the total phenolic content of the ethanolic extracts was 3 to 4 times greater than that of the ethyl acetate extracts (Table 1), emphasizing the superior effectiveness of ethanol in extracting phenolic compounds. Among the varietals, Tannat showed the highest total phenolic content, followed by Bonarda, which may be attributed to the distinct composition of the Tannat varietal (higher content of tannins, flavones/flavonols, and non-flavonoid phenolics). In contrast, the anthocyanin content in Bonarda ethanolic extracts was 1.5 to 2.6 times higher than in the other varietals. The enhanced extraction efficiency of ethanol, particularly for flavonoids and tannins, highlights its potential for producing bioactive-rich extracts with health-promoting properties. Previous studies have shown that for grape pomace, ethanol is particularly effective at recovering phenolic compounds [46,47,48], while in other plant matrices, the ethyl acetate fraction has demonstrated higher phenolic recovery [49,50].

The HPLC-DAD analysis (Table 2) allowed the identification of 16 phenolic acids, 18 flavonoids, and 3 stilbenoids in the extracts, with most of these compounds present in the three wine varietals. The ethanolic extracts generally exhibited higher compound concentrations than the ethyl acetate extracts. In general, phenolic acids were more concentrated in ethanolic extracts due to their solubility properties, with 4-*O*-caffeoylquinic acid, vanillic acid, and chlorogenic acid among the most abundant. Similar trends were observed for flavonoids, with ethanolic extracts containing higher concentrations of (+)-catechin, epicatechin, rutin, myricetin, quercetin, and phloridzin. Notably, ethyl acetate extracts had higher levels of quercetin-3-*O*-glucopyranoside than their corresponding ethanolic extracts, while kaempferol and naringin were found in comparable concentrations across both solvent systems. Between the varietals, notable quantitative differences were observed; for instance, the ethanolic extract of Tannat had higher levels of key phenolic acids, as well as catechin and rutin. On the other hand, Bonarda extracts exhibited higher concentrations of 4,5-di-*O*-caffeoylquinic acid than the other extracts. The vanillic acid content in Bonarda’s ethyl acetate extract was 2 to 2.4 times higher than that of the other ethyl acetate extracts and comparable to levels in the ethanolic extracts. Stilbenoids, such as *trans*-polydatin, resveratrol, and trans-epsilon viniferin, were detected in almost all varieties except for the ethyl acetate extract of the Malbec variety.

The antioxidant activity of the extracts was evaluated through different method-ologies (Table 3). All extracts have antioxidant capacity, mainly the ethanolic extracts, with the ethanolic extract of Tannat being the most active (lowest IC50 value). These results correlate with the concentration of phenolic compounds. Although the extracts are slightly less active than pure compounds used as standards, they have potential ap-plications in food preservation, nutraceuticals, or cosmetics, where oxidative stability is key.

The rise in antimicrobial resistance globally has emerged as a significant public health concern, necessitating the search for new antimicrobial agents. The food industry generates substantial byproducts rich in bioactive compounds, mainly phenolic compounds, which have garnered significant attention from researchers and industry due to their antioxidant and antimicrobial properties [51]. However, research on grape byproducts has largely focused on their antibiotic effects rather than their ability to counteract pathogenic mechanisms [52,53]. Only a limited number of studies have explored the antipathogenic properties of wine pomace, and in grape varieties different from those in the present study. For instance, Viola et al. [48] demonstrated that extracts from Torrontés grape pomace possess both antioxidant and antibiofilm activities against *P. aeruginosa* and *Staphylococcus aureus*. Interestingly, while Cabernet sauvignon grape pomace extracts obtained via accelerated solvent extraction exhibited *P. aeruginosa* and *Staphylococcus epidermidis* biofilm inhibition, those derived from conventional ethanolic and methanolic methods did not show significant antibiofilm effects [39]. Additionally, Sateriale et al. [54] (2024) reported that polyphenolic extracts from Aglianico grape pomace effectively inhibited biofilm formation by *S. aureus* and *Bacillus cereus*.

**Figure 1:**
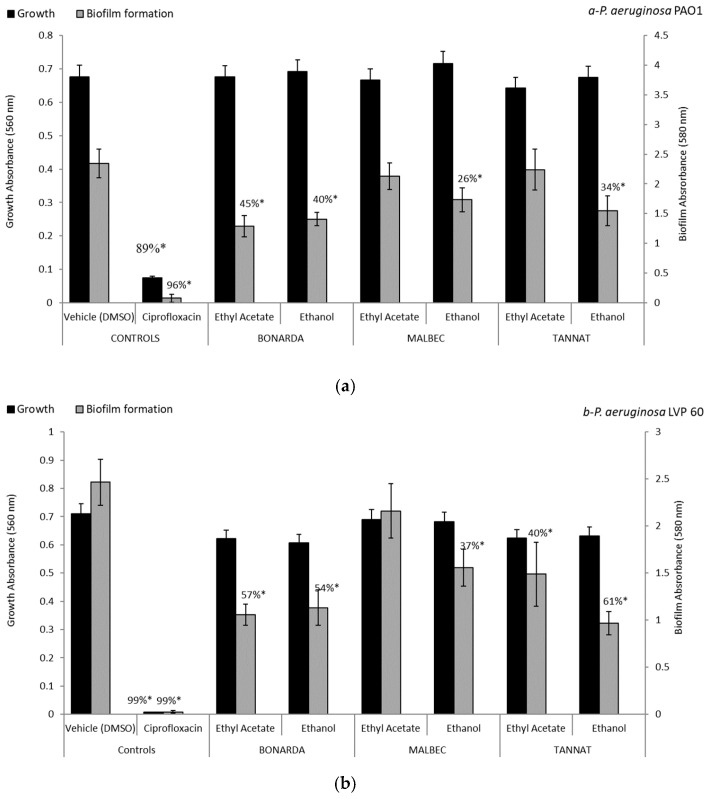
Biofilm formation and growth of *Pseudomonas aeruginosa* PAO1 (**a**) and LVP 60 (**b**) in the presence and absence of 100 μg/mL of pomace extracts and ciprofloxacin (1 μg/mL). The values represent the means ± SD. * The values are significantly different at *p* ≤ 0.05 compared to the respective DMSO control.

Regarding biofilm formation by *P. aeruginosa* (Figure 1), ethanolic extracts from the pomace of the three varietals, at a concentration of 100 µg/mL, inhibited biofilm formation in both strains: PAO1, clinical isolate, and LVP 60, contaminated water isolate. The inhibition rates were 40% and 54% for Bonarda, 26% and 37% for Malbec, and 34% and 61% for Tannat, respectively. Notably, only the ethyl acetate extract of Bonarda inhibited biofilm formation in both strains, with 45% and 57% inhibition rates. Unlike ciprofloxacin, these extracts did not inhibit bacterial growth but significantly reduced biofilm production, suggesting an antipathogenic effect that does not involve a traditional antibiotic action. This inhibition of biofilms is particularly relevant, since biofilms contribute to bacterial resistance and persistence. The ability of these extracts to reduce biofilm formation without killing bacteria could prevent the development of bacterial resistance, offering an alternative to controlling pathogenic infections.

The biological activity of many phenolic compounds has been studied individually, providing partial support for the results obtained in this study. For instance, proanthocyanidins (condensed tannins) from grape seed extract have been shown to suppress biofilm formation by *Escherichia coli* and *Salmonella Typhimurium* effectively [35]. Several natural compounds, including *p*-coumaric acid, gallic acid, ferulic acid, quercetin, caffeic acid, ursolic acid, and rutin, have been highlighted for their effectiveness in controlling *P. aeruginosa* biofilm formation [55,56]. Ivanov et al. [57] reported that rutin and ferulic acid significantly reduced biofilm formation, while catechin inhibited *P. aeruginosa* biofilm formation on surfaces [58]. Additionally, resveratrol at 500 µM partially disrupted the compact structure of *P. aeruginosa* PAO1 biofilm [59]. Kaempferol has also exhibited inhibitory effects against both Gram-positive and Gram-negative bacteria [60]. Chlorogenic acid has shown significant activity against *P. aeruginosa*, while vanillic acid displayed strong inhibitory effects against Enterobacter, with a minimum inhibitory concentration (MIC) of 800 µg/mL [61,62]. The highest biofilm attenuation was observed at twice the MIC of vanillic acid, highlighting its dose-dependent antibiofilm efficacy [63]. These compounds are believed to exert their effects through QS inhibition and the suppression of autoinducer production in *P. aeruginosa* [64].

The connection between virulence factor production and antioxidant activity is well established. The accumulation of reactive oxygen species (ROS) within cells induces oxidative stress, and biofilm development. ROS promotes microbial adhesion, enhancing biofilm formation [65]. Oxidative stress is also recognized as a critical mechanism in inflammatory processes, especially in Gram-negative infections of the intestinal mucosa [66,67]. Studies have reported the high antioxidant activity of grape pomace, indicating its potential as a source of natural antioxidants [23,36,41,48]. Moreover, the antioxidant activity correlates positively with phenolic compound concentration and antibiofilm activity. This aligns with previous findings, where methanol and ethyl acetate extracts from Torrontés wine pomace, which contained the highest total polyphenol levels, exhibited the strongest ABTS^•+^ and nitric oxide scavenging capacity, the highest Fe^3^⁺ reducing power, and the most significant biofilm inhibition. There is a direct relationship between polyphenol content, antioxidant potential, and antibiofilm properties in winemaking byproducts [48].

**Figure 2:**
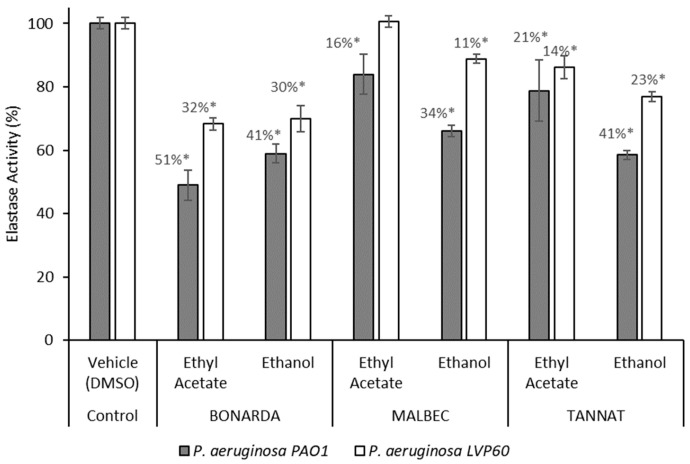
Elastase activity of *Pseudomonas aeruginosa* PAO1 and LVP 60 in the presence and absence of 100 μg/mL of pomace extracts. The values represent the means ± SD. * The values are significantly different at *p* ≤ 0.05, compared to the respective DMSO control.

The extracts also showed significant inhibition regarding elastase activity in *P. aeruginosa* (Figure 2). Elastase is a key virulence factor in *P. aeruginosa*, contributing to tissue damage and immune evasion during infections. In the PAO1 and LVP 60 strains, the extracts inhibited elastase activity in a range of 16% to 51%, with the ethanolic extracts being the most effective. Inhibition rates were 41% and 30% for Bonarda, 34% and 11% for Malbec, and 41% and 23% for Tannat, for the PAO1 and LVP 60 strains, respectively. Notably, the ethyl acetate extract of Bonarda showed significant elastase inhibition in the PAO1 strain, with an inhibition rate of 50% at a concentration of 100 µg/mL. Inhibiting elastase production can weaken the pathogenic potential of bacteria without exerting selective pressure for resistance development, as seen with conventional antibiotics, highlighting the potential of these extracts as natural alternatives for mitigating bacterial virulence in clinical or industrial settings.

**Figure 3:**
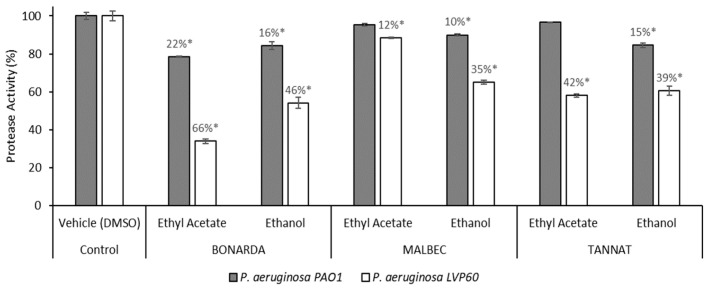
Protease activity of *Pseudomonas aeruginosa* PAO1 and LVP 60 in the presence and absence of 100 μg/mL of pomace extracts. The values represent the means ± SD. * The values are significantly different at *p* ≤ 0.05, compared to the respective DMSO control.

On the other hand, the protease activity assays demonstrated that the *P. aeruginosa* strains grew in the presence of the ethanolic extracts of the three pomace varietals, exhibiting inhibition of the protease activity in a range of 10% to 46%, with the LVP 60 strain being the most sensitive (Figure 3). Notably, Bonarda and Tannat extracts were more active than Malbec, showing stronger inhibition of protease activity. The ethyl acetate extract from Bonarda was the only one to inhibit protease activity in both strains, with inhibition rates of 22% for PAO1 and a remarkable 66% for LVP 60. The higher sensitivity of LVP 60 and the increased activity of Bonarda and Tannat suggest varietal differences in the bioactive compound profiles of the pomace extracts, which could be exploited for strain-specific therapeutic strategies. These findings highlight the potential of pomace extracts as natural virulence modulators, offering a complementary approach to traditional antimicrobial therapies by targeting bacterial virulence factors rather than promoting resistance.

The enzymes proteases and elastases, regulated by the LasR and RhlR QS systems, play a critical role in host tissue invasion and immune system evasion. Chlorogenic acid, detected in wine pomace, can inhibit elastase activity [61]. Additionally, flavonoids such as myricetin, quercetin, and kaempferol, also present in wine pomace, have demonstrated human neutrophil elastase inhibition [68,69]. An ethanolic extract from alperujo, an olive industry byproduct, at a concentration of 100 μg/mL, significantly reduced elastase activity in *P. aeruginosa* strains LVP 60 by 99% and PAO1 by 81% [70]. Resveratrol (250 µM) and quercetin (500 µM) inhibited proteolytic activity in *P. aeruginosa* by 35.9% and 34.0%, respectively [71]. Quercetin significantly inhibited protease production by *P. aeruginosa* PAO1 (43%) Ouyang et al. [72].

**Figure 4:**
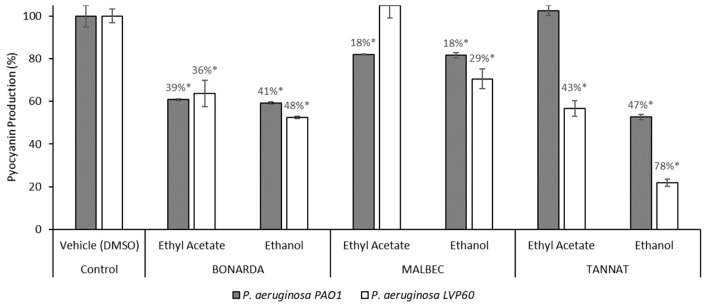
Pyocyanin production by *Pseudomonas aerguninosa* PAO1 and LVP 60 in the presence and absence of 100 μg/mL of pomace extracts. The values represent the means ± SD. * The values are significantly different at *p* ≤ 0.05, compared to the respective DMSO control.

Pyocyanin is recognized as one of the most significant virulence factors in *P. aeruginosa*. It plays a crucial role in its pathogenicity and exhibits antimicrobial properties against various bacterial and fungal species [73]. Consequently, targeting the inhibition of this virulence factor serves as a valuable indicator of a compound’s efficacy as a QSI [59,71]. The extracts effectively inhibited the pigment pyocyanin, another virulence factor produced by *P. aeruginosa* (Figure 4). Ethanolic extracts outperformed ethyl acetate extracts in reducing pyocyanin production in both bacterial strains, with inhibition levels ranging from 18% to 78%. The Tannat ethanolic extract showed the most potent effect, decreasing pyocyanin production by 47% in PAO1 and 78% in LVP 60. This was followed by the Bonarda ethanolic extract, which achieved 41% inhibition in PAO1 and 48% in LVP 60. Among the ethyl acetate extracts, Bonarda pomace was the most effective, inhibiting pyocyanin production by 48% in PAO1 and 36% in LVP 60. Ugurlu et al. [74] demonstrated that cinnamic acid, vanillic acid, ferulic acid, and caffeic acid reduced pyocyanin production by 9–21% at sub-MIC, while Ouyang et al. [72] reported that quercetin inhibited pyocyanin production by 58% at 53 μM. Likewise, chlorogenic acid has been shown to reduce pyocyanin production [61].

Swarming motility, closely linked to virulence and antibiotic resistance, is another adaptive mechanism contributing to biofilm formation and infection persistence in various microorganisms [75,76]. All extracts attenuated swarming motility in both *P. aeruginosa* strains, with inhibition ranging from 17% to 68% (Table 4). This reduction in swarming motility suggests that the extracts may interfere with QS mechanisms, which are crucial for regulating collective behaviors in *P. aeruginosa*. Cranberry proanthocyanidins and other tannins have been reported to completely inhibit *P. aeruginosa* swarming motility [77]. Gallic acid and naringenin inhibited swarming by more than 20%, and quercetin reached 50%, while resveratrol had no significant effect [71]. In contrast, Nunes Sagini et al. [39] informed that grape extract of Cabernet sauvignon increased *P. aeruginosa* P14 swarming.

**Figure 5:**
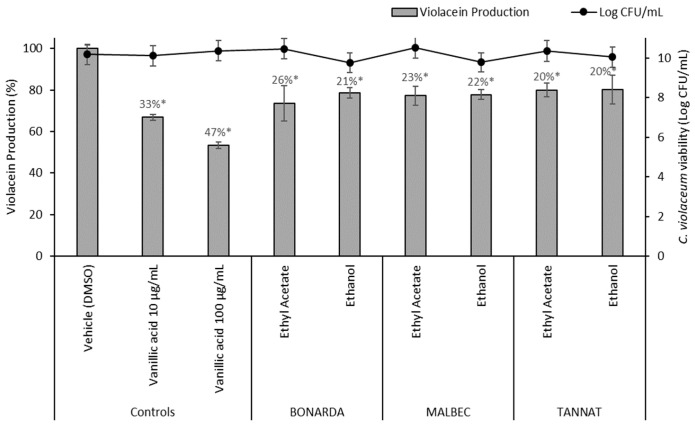
Violacein production by *Chromobacterium violaceum* CECT 494 in the presence of 100 μg/mL of pomace extracts and the controls (DMSO and vanillic acid). The values represent the means ± SD. * The values are significantly different at *p* ≤ 0.05, compared to the DMSO.

**Figure 6:**
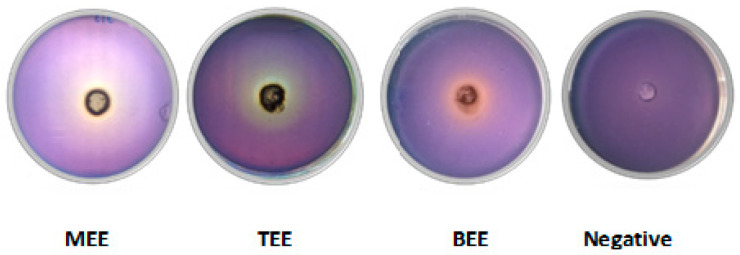
Quorum sensing biosensor assay with Chromobacterium violaceum CV026 performed with wine pomace extracts at 5 mg/well (MEE: Malbec ethanolic extract, TEE: Tannat ethanolic extract, and BEE: Bonarda ethanolic extract) and DMSO (negative control).

Altering QS can reduce biofilm formation, virulence factor production, and antibiotic resistance. Two assays were conducted to evaluate anti-QS activity. The *Chromobacterium violaceum* CECT 494 assay detects inhibitors of AHL synthesis, while the *C. violaceum* CV026 assay identifies quenchers of AHL signals. In the *C. violaceum* CECT 494 biosensor assay, pomace extracts reduced violacein production 20–26% without affecting bacterial growth (Figure 5). Vanillic acid, a known QS inhibitor present at varying concentrations in the extracts, served as a positive control, showing inhibition rates of 33% and 47% at 10 and 100 µg/mL, respectively. Additionally, wine pomace extracts competitively inhibited the interaction between AHL and its receptor CviR, impeding violacein production. This effect was evidenced by cloudy, colorless areas observed around the wells containing extracts (Figure 6).

Resveratrol has demonstrated the ability to inhibit QS, reducing violacein production in *C. violaceum* ATCC 12472 by 60% at 6.0 μM without affecting bacterial growth. In contrast, gallic acid and phloridizin did not impact violacein production [78]. Similarly, Duarte et al. [79] reported violacein inhibition by resveratrol, suggesting that its anti-QS activity is related to mimicking QS signals, thereby disrupting bacterial communication. Bali et al. [80] also found that gallic acid did not exhibit anti-QS activity in the biosensor strain. However, tests using only wild-type *C. violaceum* strains are insufficient to fully understand the QS inhibitory mechanism, as violacein inhibition may result from reduced autoinducer production (inhibition of AHL synthesis) or interference with the AHL-dependent transcriptional activator [66]. Thus, besides *C. violaceum* ATCC 12472, additional assays with strains like *C. violaceum* CV026, a mutant unable to produce AHLs but capable of responding to exogenous AHLs, may be necessary [81].

To evaluate the safety of the extracts, an acute toxicity test was performed using *Artemia salina* (a complex organism). The ethanolic extracts from Bonarda, Malbec, and Tannat wine pomace showed no signs of toxicity at concentrations up to 1000 μg/mL. However, the ethyl acetate extracts could not be tested due to solubility issues that hindered proper dilution. As reported by Nguta et al. [82], extracts with LD_50_ values (the concentration that kills 50% of *A. salina* larvae) above 1000 μg/mL are classified as non-toxic.

It is well established that the distribution and concentration of total polyphenolic compounds, as well as specific polyphenolic constituents, vary considerably across *Vitis vinifera* cultivars [83]. In this study, the ethanolic extract of Tannat showed superior antioxidant properties in all assays, along with greater inhibition of *P. aeruginosa* biofilm formation, pyocyanin production, and swarming motility compared to the other extracts. It also demonstrated elastase and protease inhibition levels comparable to the Bonarda extract and higher than those of Malbec. Furthermore, all extracts attenuated QS similarly. These findings may be correlated with the higher levels of total phenolics, flavones/flavonols, non-flavonoids, and tannins in the Tannat extract, supporting the link between phenolic content, antioxidant capacity, and the antipathogenic potential of wine pomace extracts.

Pure phenolic compounds are not approved as food preservatives; however, botanical extracts rich in polyphenols can be incorporated into perishable foods [84]. Given that polyphenols from wine pomace offer a natural and safe alternative for extending the shelf life of food products by preventing oxidation and spoilage caused by pathogens, the present findings highlight the potential of grape pomace as a food preservative. Grape pomace powder or extracts have been successfully used as preservatives in cheeses, yogurts, and gelatin, enhancing their stability [85,86,87,88,89]. Additionally, grape seed extract has been applied to fresh fish and fish products, effectively reducing lipid oxidation and inhibiting microbial growth [89,90,91,92].

## 3. Materials and Methods

### 3.1. Sampling and Extraction

The studies were performed with wine pomace, *Vitis vinifera* L., cv. Malbec, cv. Bonarda, and cv. Tannat obtained from wineries located in Cafayate, Salta, Argentina. Fresh wine pomace samples were collected and placed in ice-cooled boxes during transportation to the laboratory, where they were stored at –20 °C until processing.

The solid–liquid extraction method employed was maceration (1 kg dry pomace/L) by agitation for 24 h at 25 °C. To obtain extracts containing various classes of compounds, solvents with different polarities were used. The extraction process was carried out successively, beginning with ethyl acetate (moderate polarity), followed by ethanol 96° (high polarity), until exhaustion. This approach allowed for the selective extraction of different groups of bioactive compounds based on their solubility in each solvent.

After extraction, the resulting extracts were filtered using a Whatman Nº1 filter paper (Cytiva, Piscataway, NJ, USA) and concentrated through vacuum evaporation with a rotary evaporator (Rotavapor Buchi R-300, BUCHI, Flawil, Switzerland) at 30 °C. The dry extracts (DW) were then re-suspended in dimethyl sulfoxide (DMSO) for subsequent phytochemical and biological analyses.

### 3.2. Phytochemical Analysis

#### 3.2.1. Determination of Total Phenolic and Nonflavonoid Compounds

The total phenolic content of grape pomace extracts was determined using the Folin–Ciocalteu reagent (Biopack, Buenos Aires, Argentina), following the method described by Viola et al. (2018) [48]. Gallic acid was used as the reference standard to create a calibration curve, and the results were expressed as µg gallic acid equivalents per mg of dry weight (µg GAE/mg). Nonflavonoid phenolics were quantified by measuring the remaining total phenolic content in the supernatant after the precipitation of flavonoids with acidic formaldehyde [48]. The results were expressed as µg GAE/mg.

#### 3.2.2. Determination of Flavones/Flavonols

Flavones/Flavonols were determined following the procedure reported by Tapia et al. [23]. Samples were reacted with AlCl_3_, and the absorbance was measured at 420 nm. The amount of flavonoid was calculated using a linear regression equation obtained from a quercetin calibration curve. The flavonoid content was reported as µg quercetin equivalents per mg of dry weight (µg QE/mg).

#### 3.2.3. Determination of Anthocyanins

The assessment of the total anthocyanin content was carried out using the pH differential method, and the results were expressed as µg of cyanidin-3-glucoside equivalents per mg of extract (µg C3GLE/mg) according to Tapia et al. [23].

#### 3.2.4. Determination of Condensed Tannins

The total condensed tannins content was determined using 4-dimethylaminocinnamaldehyde (DMAC), as described by Viola et al. [48]. The absorbance was measured at 640 nm using a spectrophotometer. Proanthocyanidin B2 was used as the standard drug, and the results were expressed as µg of proanthocyanidin B2 equivalents per mg of dry weight (µg PB_2_E/mg).

#### 3.2.5. Identification of Phenolic Compounds Using HPLC-DAD

Phenolic compounds were identified using a Shimadzu HPLC system (Kyoto, Japan) equipped with a Gemini C18 column (Phenomenex, Torrance, CA, USA) (250 × 4.6 mm, 5 μm) at 25 °C, following the method described by Moreira et al. [93]. The solvent system used was methanol (A) and water (B), both acidified with 0.1% formic acid. The gradient program ranged from 20% to 100% A over 100 min, with reconditioning phases before each injection. Detection was performed at 280, 320, and 360 nm, depending on the phenolic compound’s absorption maxima. Extracts were dissolved in methanol/water (20:80) and filtered before injection. Quantification was based on calibration curves of pure standards, expressed as mg per 100 g of dry extract.

### 3.3. Antioxidant Activity Assays

#### 3.3.1. Total Antioxidant Activity

Pomace extracts were tested spectrophotometrically at 695 nm for their total antioxidant activity following the technique described by Tapia et al. [23]. Briefly, the reaction mixture, containing 1000 µL of a 4 nM ammonium molybdate solution and 200 µg DW/mL of pomace extract, was left to react in a water bath for 90 min at 95 °C. Ascorbic acid was used to plot the standard curve (R^2^ = 0.9903, *p* ≤ 0.05), and results were expressed as micrograms of ascorbic acid equivalents per mg of dry weight (µg AAE/mg DW).

#### 3.3.2. ABTS^•+^ Free Radical Scavenging Activity

Pomace extracts were evaluated for their capacity to scavenge ABTS^•+^ radical [48]. Concentrations of 5–100 µg DW/mL of orujo extracts reacted with an ABTS^•+^ solution (absorbance of 0.7 at 734 nm); while Trolox (2.5–7.5 µg/mL) were used as positive controls. The IC_50_ (the concentration necessary to scavenge 50% of the ABTS^•+^ free radicals) was determined by linear regression analysis plotted with the percentage of scavenging obtained from the absorbance read at 734 nm after 6 min of incubation.

#### 3.3.3. Nitric Oxide Scavenging Activity

Pomace extracts (100–1000 μg DW/mL) were put to react with sodium nitroprusside (100 mM) and Griess reagent (Sigma-Aldrich, St. Louis, MO, USA) to determine their capacity to depurate nitric oxide at 550 nm following the technique described by Viola et al. [48]. Ascorbic acid (25–400 µg/mL) was used as a positive control. To determine the concentration required to scavenge 50% of the nitric oxide radical (IC_50_), a regression curve was constructed by plotting the scavenging concentration against the sample concentration.

#### 3.3.4. Iron III Reducing Power

The capacity of orujo extracts (100–500 µg DW/mL) to reduce Fe^3+^ to its ferrous form was determined spectrophotometrically at 700 nm according to Viola et al. [48]. BHT (3–13 µg/mL) was used as a positive control. The concentration that reduces 50% of the Fe^3+^ (RC_50_) was determined by linear regression analysis.

### 3.4. Antipathogenic Analysis

#### 3.4.1. Bacterial Strains

Two strains of *P. aeruginosa* (PAO1 and LVP 60) were used as models for studying virulence of Gram-negative bacteria. The strain PAO1 isolated from infected wounds is widely used for research on opportunistic pathogens [94] and the strain LVP 60 was isolated from drinking water samples [95].

The strains used for the anti-quorum sensing (anti-QS) tests were *Chromobacterium violaceum* CECT 494 (wild type) and *C. violaceum* CV026 (mutant), both obtained from the Spanish Type Culture Collection (CECT) in Valencia, Spain.

The strains were activated from frozen stocks stored at −80 °C in Luria–Bertani (LB) broth with 20% glycerol for *P. aeruginosa* strains, and in LB Tryptein broth with 20% glycerol for *C. violaceum* strains. The revival process involves two successive passages of the frozen cultures in fresh broth at 37 °C to ensure optimal activation.

#### 3.4.2. Bacterial Growth

In a 96-well microtiter polystyrene plate, 195 µL of an overnight culture of *P. aeruginosa* grown in Luria–Bertani (LB) medium diluted to reach the appropriate inoculum (OD 560 nm:0.1) was mixed with 5 µL of pomace extracts’ solutions (100 µg/mL final concentration), and incubated statically for 24 h. Bacterial growth was detected as turbidity (560 nm) using a microplate spectrophotometer (MultiskanGo, Thermo Fisher Scientific, Waltham, MA, USA).

Inhibition of bacterial growth mediated by the pomace was assessed by comparison with bacterial growth in the control wells containing 2.5% DMSO. Ciprofloxacin at 1 µg/mL was incorporated into the bioassay as a negative control.

#### 3.4.3. Biofilm Formation Assay

The biofilm quantification was studied as described by Viola et al. [48]. Biofilms formed after 24 h incubation of bacterial cultures prepared as described in the previous paragraph were stained with 200 µL of an aqueous solution of crystal violet (0.05%, *w*/*v*) for 20 min, two washes with water removed unbound stains, and dried crystal violet bound to biofilm was solubilized with 200 µL acetic acid 30% during 30 min at 37 °C with shaking. Absorbance at 580 nm of crystal violet solution was determined using a microplate spectrophotometer (MultiskanGo, Thermo Fisher Scientific, Waltham, MA, USA). The following formula calculated biofilm inhibition (%):

Biofilm inhibition (%) = [(Control OD595 nm − Experimental OD595 nm)/Control OD595 nm] × 100.

#### 3.4.4. Elastase Activity

Elastase activity was evaluated using an elastin-Congo red conjugate, a substrate specific for elastase, as described by Viola et al. [70]. In summary, 500 μL of a 5 mg/mL substrate solution dissolved in Tris-HCl buffer (pH 8.0) was combined with 500 μL of overnight culture supernatant from *P. aeruginosa* grown in Mueller-Hinton (MH) medium, with and without the extracts (final concentration 100 µg/mL). The mixture was incubated for 24 h at 37 °C and 150 rpm. Post-incubation, the samples were centrifuged at 10,000 rpm for 10 min, and the absorbance of the supernatant was measured at 495 nm using a microplate reader. A control assay was performed concurrently using a mixture of MH medium-Tris-HCl buffer (1:1). Elastase inhibition (%) was calculated by the following formula:

Enzyme inhibition (%) = [(Control OD495 nm -Experimental OD494 nm)/Control OD495 nm] × 100.

#### 3.4.5. Protease Activity

To assess azocasein proteolytic activity, *P. aeruginosa* strains were incubated with and without pomace extracts at 37 °C for 48 h. Proteolytic activity in the cell-free supernatant was measured using the method of Gupta et al. [96]. In brief, 100 μL of supernatant from treated or untreated cultures was mixed with 400 μL of 0.3% azocasein in 0.05 M Tris-HCl (pH 7.5) and incubated at 37 °C for 1 h. The reaction was stopped with 10% trichloroacetic acid and centrifugation at 3500× *g* for 5 min. The absorbance of the clear supernatant was measured at 420 nm. Protease inhibition (%) was calculated by the following formula:

Enzyme inhibition (%) = [(Control OD420 nm − Experimental OD420 nm)/Control OD420 nm] × 100.

#### 3.4.6. Pyocyanin Quantification

The quantification of pycyanin was carried out according to the method described by Díaz et al. [97]. Nine milliliters of cell-free supernatant from both treated and untreated cultures were extracted with 9 mL of chloroform (mixed for 15 s and left to stand to allow phase separation). The entire chloroform phase (light blue/turquoise in color) was collected, and 1 mL of 0.2 M HCl was added, followed by vortexing for approximately 15 s and then allowing it to rest. A 200 µL aliquot of the upper phase (turned pink/fuchsia), was taken and placed in a 96-well microplate to measure the absorbance at 520 nm.

Pyocyanin inhibition (%) = [(Control OD520 nm − Experimental OD520 nm)/Control OD520 nm] × 100.

#### 3.4.7. Swarming Motility

The effect of pomace extracts on the motility of *P. aeruginosa* strains was assessed using the methodology described by Viola et al. [70]. Each pomace extract was mixed in two concentrations (250 µg/mL and 500 µg/mL), with swarm agar (0.5%) medium. Then, each *P. aeruginosa* strain was point inoculated and incubated at 37 °C for 24 h. DMSO was used as a negative control (1%, *v*/*v*). The effect of pomace extracts on swarming motility was determined by measuring circular turbid zones in comparison with those in the control. The motility measurements were made using Image J 1.47 V software.

#### 3.4.8. Bioassay for the Detection of Anti-QS Activity

For the *C. violaceum* CECT 494 assay, violacein production, regulated by AHL autoinducers, was quantified in LB cultures treated with extracts (100 µg/mL), vanillic acid (10 and 100 µg/mL), or DMSO (control). After 24 h of incubation at 28 °C, violacein was extracted and its concentration was measured by absorbance at 585 nm. Bacterial viability was evaluated through serial dilution plating [70].

In the *C. violaceum* CV026 assay, violacein production is dependent on the exogenous addition of C6-HSL (short-chain AHLs). Agar plates supplemented with C6-HSL were inoculated with *C. violaceum* CV026, and wells loaded with extracts were examined for QS inhibition, indicated by a colorless zone surrounding the wells [70].

### 3.5. Toxicity Assay

The acute toxicity levels of pomace extracts, with concentrations from 250 to 1000 μg/mL, were evaluated using the *Artemia salina* test [23]. The negative control wells contained DMSO to a final concentration lower than 0.3%. Survival percentages were calculated by comparing the number of survivors in the test wells with respect to the negative control.

### 3.6. Statistical Analysis

All data are expressed as mean value ± SD of triplicate and octuplicate samples in phytochemical analysis and microbiological test, respectively. Statistical significance was analyzed using Tukey’s *t*-test at *p* < 0.05 (software InfoStat, Student Version, 2020), considering a confidence level of 95%.

## 4. Conclusions

Ethanolic extracts of wine pomace, particularly those derived from Tannat, exhibited strong antioxidant and antipathogenic activities by effectively inhibiting key virulence factors in *P. aeruginosa*. These results suggest that such extracts have potential as natural alternatives for combating bacterial contamination and mitigating the risk of resistance development.

However, while the in vitro antipathogenic effects are promising, further research is needed to assess their efficacy in real-world applications. In particular, their potential as biofilm inhibitors on various surfaces within the food industry and on food packaging where contamination is a major concern. Additionally, future studies should expand the range of pathogens tested to fully elucidate the antimicrobial potential of these natural extracts.

## Figures and Tables

**Table 1:** The polyphenol composition of the extracts.

Phytochemical Composition	Ethyl Acetate			Ethanol		
	Bonarda	Malbec	Tannat	Bonarda	Malbec	Tannat
Total phenolics (µg GAE/mg)	37.8 ± 3.3 ^b^	23.8 ± 3.3 ^a^	39.9 ± 3.3 ^b^	113.8 ± 6.6 ^d^	97.3 ± 6.6 ^c^	162.5 ± 6.6 ^e^
Non Flavonoids (µg GAE/mg)	20.4 ± 2.6 ^b^	14.6 ± 0.3 ^a^	18.2 ± 1.2 ^a,b^	40.5 ± 0.2 ^d^	26.1 ± 3.0 ^c^	53.8 ± 3.1 ^e^
Flavones/Flavonols (µg QE/mg)	3.6 ± 0.3 ^a,b^	3.4 ± 0.2 ^a,b^	5.4 ± 0.1 ^d^	4.8 ± 0.2 ^c^	4.0 ± 0.6 ^b,c^	7.0 ± 0.3 ^e^
Tannins (µg PB_2_E/mg)	1.1 ± 0.0 ^a^	0.9 ± 0.0 ^a^	1.3 ± 0.0 ^a^	24.5 ± 0.3 ^c^	10.6 ± 0.0 ^b^	44.4 ± 1.0 ^d^
Anthocyanins (µg C3GE/mg)	ND	ND	ND	2421.3 ± 11.8 ^c^	922.6 ± 76.7 ^a^	1632.3±431.1 ^b^

GAE: gallic acid equivalents. QE: quercetin equivalents. PB_2_E: proanthocyanidin B2 equivalents. C3GE: cyanidin-3-glucoside equivalents. ND: not determined due to solubility problems. The values are reported as mean ± S.D. Different letters in the same row show significant differences among each treated group according to Tukey’s test (*p* < 0.05).

**Table 2:** Phenolic compounds quantified (mg/100 g DW) in wine pomace extracts through HPLC-DAD.

Phenolic Compound	Ethyl Acetate			Ethanol		
	Bonarda	Malbec	Tannat	Bonarda	Malbecc	Tannat
Phenolic acids						
Gallic acid	5.5 ± 0.3	402.6 ± 20.1	503.3 ± 25.2	4.8 ± 0.2	107.3 ± 5.4	143.6 ± 7.2
Protocatechuic acid	59.00 ± 3.0	13.0 ± 0.7	4.8 ± 0.2	8.8 ± 0.4	149.7 ± 7.5	ND
Neochlorogenic acid	2.19 ± 0.1	<LOD	1.2 ± 0.1	25.6 ± 1.3	62.3 ± 3.1	71.2 ± 3.6
Caftaric acid	32.8 ± 1.6	5.4 ± 0.3	21.9 ± 1.1	25.3 ± 1.3	48.3 ± 2.4	71.0 ± 3.6
Chlorogenic acid	36.8 ± 1.8	13.7 ± 0.7	30.5 ± 1.5	179.4 ± 9.0	294.4 ± 14.7	409.1 ± 20.5
4-*O*-Caffeyolquinic acid	483.5 ± 24.2	158.7 ± 7.9	913.4 ± 45.7	3980.4 ± 199.0	3340.5 ± 167.0	6431.9 ± 321.6
Vanillic acid	1604.7 ± 80.2	801.1 ± 40.1	678.8 ± 33.9	1247.8 ± 62.4	1361.7 ± 68.1	1589.3 ± 79.5
Caffeic acid	13.9 ± 0.7	10.8 ± 0.5	17.5 ± 0.9	38.3 ± 1.9	23.6 ± 1.2	57.1 ± 2.9
Syringic acid	26.3 ± 1.3	ND	ND	ND	ND	50.5 ± 2.5
*p*-Coumaric acid	12.7 ± 0.6	17.4 ± 0.9	13.5 ± 0.7	8.2 ± 0.4	12.1 ± 0.6	8.7 ± 0.4
Ferulic acid	ND	<LOQ	2.1 ± 0.1	6.9 ± 0.3	9.9 ± 0.5	25.2 ± 1.3
Caffeine	16.5 ± 0.8	13.5 ± 0.7	7.7 ± 0.4	24.3 ± 1.2	31.2 ± 1.6	14.8 ± 0.7
Sinapic acid	ND	ND	1.6 ± 0.1	ND	ND	22.8 ± 1.1
3,5-di-Caffeoylquinic acid	1.2 ± 0.1	4.9 ± 0.2	5.2 ± 0.3	8.7 ± 0.4	12.8 ± 0.6	3.2 ± 0.2
Ellagic acid	7.4 ± 0.4	1.5 ± 0.1	4.9 ± 0.2	76.9 ± 3.8	38.4 ± 1.9	63.9 ± 3.2
4,5-di-*O*-Caffeoylquinic acid	62.1 ± 3.1	18.6 ± 0.9	25.4 ± 1.3	82.1 ± 4.1	42.3 ± 2.1	48.6 ± 2.4
Flavonoids						
(+)-Catechin	73.9 ± 3.7	31.1 ± 1.6	95.9 ± 4.8	442.8 ± 22.1	330.3 ± 16.5	720.0 ± 36.0
(−)-Epicatechin	11.8 ± 0.6	26.1 ± 1.3	13.9 ± 0.7	416.8 ± 20.8	402.2 ± 20.1	442.9 ± 22.1
Naringin	19.7 ± 1.0	20.5 ± 1.0	26.9 ± 1.3	30.4 ± 1.5	16.0 ± 0.8	22.1 ± 1.1
Quercetin-3-*O*-galactoside	22.1 ± 1.1	35.9 ± 1.8	23.8 ± 1.2	54.3 ± 2.7	27.3 ± 1.4	49.3 ± 2.5
Quercetin-3-*O*-glucopyranoside	12.0 ± 0.6	31.6 ± 1.6	2.7 ± 0.1	7.9 ± 0.4	1.4 ± 0.1	1.2 ± 0.1
Rutin	29.8 ± 1.5	6.1 ± 0.3	110.2 ± 5.5	307.5 ± 15.4	33.8 ± 1.7	832.8 ± 41.6
Phloridzin	28.3 ± 1.4	19.9 ± 1.0	20.8 ± 1.0	147.0 ± 7.4	73.0 ± 3.7	143.8 ± 7.2
Myricetin	17.3 ± 0.9	65.7 ± 3.3	92.9 ± 4.6	262.5 ± 13.1	105.9 ± 5.3	161.6 ± 8.1
Kaempferol-3-*O*-glucoside	ND	25.2 ± 1.3	52.6 ± 2.6	ND	ND	ND
Kaempferol-3-*O*-rutinoside	ND	ND	ND	ND	11.4 ± 0.6	13.2 ± 0.7
Isorhamnetin-3-*O*-rutinoside	2.4 ± 0.1	12.0 ± 0.6	10.6 ± 0.5	16.2 ± 0.8	19.7 ± 1.0	18.7 ± 0.9
Naringenin	<LOD	7.3 ± 0.4	8.4 ± 0.4	11.4 ± 0.6	8.2 ± 0.4	20.2 ± 1.0
Quercetin	1.9 ± 0.1	12.8 ± 0.6	23.2 ± 1.2	27.5 ± 1.4	47.8 ± 2.4	135.4 ± 6.8
Phloretin	2.4 ± 0.1	1.3 ± 0.1	2.8 ± 0.1	6.9 ± 0.3	1.3 ± 0.1	7.0 ± 0.4
Tiliroside	ND	2.4 ± 0.1	4.7 ± 0.2	20.1 ± 1.0	25.3 ± 1.3	23.2 ± 1.2
Kaempferol	9.0 ± 0.4	1.1 ± 0.1	7.9 ± 0.4	13.0 ± 0.7	10.9 ± 0.5	8.8 ± 0.4
Apigenin	1.1 ± 0.1	<LOD	<LOD	2.6 ± 0.1	1.4 ± 0.1	1.6 ± 0.1
Chrysin	<LOD	<LOD	<LOD	1.1 ± 0.1	1.4 ± 0.1	1.1 ± 0.1
Stilbenoids and others						
*trans*-Polydatin	2.3 ± 0.1	2.8 ± 0.1	2.5 ± 0.1	11.4 ± 0.6	1.3 ± 0.1	15.5 ± 0.8
Resveratrol	3.5 ± 0.2	<LOD	1.3 ± 0.1	7.60.4	11.5 ± 0.6	7.8 ± 0.4
*trans*-Epsilon viniferin	3.7 ± 0.2	<LOD	12.6 ± 0.6	15.0 ± 0.8	1.4 ± 0.1	20.4 ± 1.0
Total (mg/100 g DW)	2601.7	1763.2	2732.6	7504.7	6664.5	11,637.2

The results are expressed as mean ± SD; LOQ: limit of quantification; LOD: limit of detection; ND: not detected.

**Table 3:** The antioxidant capacity of the wine pomace extracts.

Variety	Extract	Phosphomolybdenum Reducing Capacity (μg AAE/mg DW)	ABTS^•+^ Scavenging IC_50_ (μg/mL)	Fe^3+^ Reducing RC_50_ (μg/mL)	NO Scavenging IC_50_ (μg/mL)
Bonarda	Ethyl acetate	27.2 ± 0.7 ^b,c^	48.4 ± 2.3 ^c^	161.2 ± 1.8 ^e^	-
	Ethanol	82.9 ± 2.6 ^e^	41.4 ± 0.1 ^b,c^	42.1 ± 0.4 ^c^	-
Malbec	Ethyl acetate	20.5 ± 1.3 ^a,b^	86.5 ± 1.5 ^f^	312.4 ± 2.9 ^f^	-
	Ethanol	66.6 ± 4.9 ^d^	71.3 ± 4.1 ^e^	67.9 ± 0.8 ^d^	-
Tannat	Ethyl acetate	27.7 ± 0.1 ^b,c^	59.9 ± 1.1 ^d^	185.4 ± 0.01 ^e^	-
	Ethanol	127.7 ± 1.8 ^f^	36.9 ± 1.9 ^b^	30.9 ± 0.01 ^b^	800.3 ± 27.4 ^b^
Controls	Trolox	-	3.7 ± 0.1 ^a^	-	-
	BHT	-	-	11.4 ± 0.1 ^a^	-
	Ascorbic acid	-	-	-	133.8 ± 6.3 ^a^

Different letters in the same column show significant differences among each treated group, according to a Tukey test (*p* ≤ 0.05). The phosphomolybdenum-reducing capacity is expressed as micrograms of ascorbic acid equivalents per milligram of dry extract (μg AAE/mg DW). The Fe^3+−^reducing capacity (RC), ABTS radical cation (ABTS^•+^) and nitric oxide (NO), and scavenging capacities (IC) are determined through linear regression analysis.

**Table 4:** Migration (swarming) percentage of *P. aeruginosa*.

Variety	Extract	*P. aeruginosa* PAO1		*P. aeruginosa* LVP 60	
		250 µg/mL	500 µg/mL	250 µg/mL	500 µg/mL
Bonarda	Ethyl Acetate	83 ± 6 ^b^(17%)	66 ± 8 ^b^(34%)	64 ± 5 ^b^(36%)	63 ± 7 ^b^(37%)
	Ethanol	52 ± 4 ^c^(48%)	40 ± 5 ^c^(60%)	65 ± 5 ^b^(35%)	52 ± 6 ^b,c^(48%)
Malbec	Ethyl Acetate	53 ± 1 ^c^(47%)	33 ± 6 ^c,d^(67%)	46 ± 4 ^c^(54%)	40 ± 3 ^c^(60%)
	Ethanol	50 ± 4 ^c^(50%)	35 ± 5 ^c,d^(65%)	42 ± 9 ^c^(58%)	43 ± 5 ^c^(57%)
Tannat	Ethyl Acetate	55 ± 3 ^c^(44%)	33 ± 2 ^c,d^(67%)	50 ± 5 ^c^(50%)	45 ± 3 ^c^(55%)
	Ethanol	54 ± 4 ^c^(46%)	32 ± 1 ^c,d^(68%)	50 ± 3 ^c^(50%)	40 ± 7 ^c^(60%)
Control	Vehicle (DMSO)	100 ± 7 ^a^	100 ± 7 ^a^	100 ± 4 ^a^	100 ± 4 ^a^

Different letters in the same column show significant differences among each treated group, according to Tukey’s test (*p* < 0.05).

## Data Availability

Data will be made available on request.
